# Supplementary material for: Sourdough Fermented Breads are More Digestible than Those Started with Baker’s Yeast Alone: An In Vivo Challenge Dissecting Distinct Gastrointestinal Responses
Source: Nutrients. 2019 Dec 4;11(12):2954. doi: 10.3390/nu11122954 (PMC6950244; doi:10.3390/nu11122954)
Supplement: Supplementary file 1 [file nutrients-11-02954-s001.pdf]

Table S1. Proximal composition and energy value of the experimental breads: BYB, leavened with baker's yeast 1.5% for 90 min at 30 °C; SB, sourdough bread leavened with S_4 (20%) and baker's yeast 1.5% for 90 min at 30°C; t-SB, sourdough bread leavened with S_{24} (20%) for 4 h at 30 °C. .

	**BYB**	**SB**	**t-SB**
Energy value (kj/100g)	1201 ± 52 [a]	1194 ± 57 [a]	1176 ± 51 [a]
Total carbohydrates (% of d.m.)	84.5 ± 0.2 [a]	83.8 ± 0.3 [a]	83.5 ± 0.2 [a]
Dietary Fibre (% of d.m.)	3.10 ± 0.07 [a]	3.05 ± 0.07 [a]	3.18± 0.08 [a]
Lipids (% of d.m.)	1.3 ± 0.1 [a]	1.2 ± 0.2 [a]	1.2 ± 0.1 [a]
Proteins (% of d.m.)	10.9 ± 0.5 [a]	10.8 ± 0.6 [a]	11.1 ± 0.4 [a]
Ash (% of d.m.)	0.5 ± 0.1 [a]	0.4 ± 0.1 [a]	0.5 ± 0.1 [a]

d.m.: dry matter, The data are the means of three independent experiments ± standard deviations (n = 3).Values in the same row with different superscript letters differ significantly ($p < 0.05$).

Table S2. Gastrointestinal motility: data resulting from the study of the gastric and gallbladder emptying and H_2 breath technique. BYB, leavened with baker's yeast 1.5% for 90 min at 30 °C; SB, sourdough bread leavened with S_4 (20%) and baker's yeast 1.5% for 90 min at 30 °C; t-SB, sourdough bread leavened with S_{24} (20%) for 4 h at 30 °C. NU, Nutridrink.

		**BYB**	**SB**	**t-SB**	**NU**
Gastric emptying					
Basal antral area	cm^2	4.3 ± 0.3 ^{a}	4.0 ± 0.2 ^{a}	4.2 ± 0.1 ^{a}	4.1 ± 0.2 ^{a}
Max. postprandial antral area	cm^2	11.4 ± 0.4 ^{a}	11.7 ± 0.5 ^{a}	11.4 ± 0.5 ^{a}	10.7 ± 0.4 ^{a}
Residual antral area	cm^2	4.7 ± 0.2 ^{a}	4.2 ± 0.1 ^{b}	4.2 ± 0.1 ^{b}	4.8 ± 0.2 ^{a}
AUC	cm^2 × 120 min	854 ± 119 ^{a}	774 ± 105 ^{a}	758 ± 105 ^{b}	765 ± 19.1 ^{a}
Half-emptying time	min	43.7 ± 4.4 ^{a}	34.8 ± 2.4 ^{b}	30.8 ± 2.3 ^{b}	29.0 ± 1.9 ^{b}
Emptying rate	cm^2/min	−1.20 ± 0.1 ^{a}	−0.28 ± 0.1 ^{c}	−0.24 ± 0.0 ^{c}	−0.66 ± 0.0 ^{b}
Gallbladder emptying					
Fasting vol.	mL	18.9 ± 1.3 ^{a}	18.6 ± 1.1 ^{a}	17.6 ± 1.2 ^{a}	18.6 ± 0.9 ^{a}
Ejection vol.	mL	8.1 ± 0.6 ^{b}	7.3 ± 0.7 ^{b}	6.3 ± 0.7 ^{b}	12.8 ± 0.9 ^{a}
Residual vol.	mL	10.8 ± 1.1 ^{a}	11.3 ± 0.8 ^{a}	11.2 ± 1.2 ^{a}	5.9 ± 0.7 ^{b}
Time to residual vol.	min	72.5 ± 3.6 ^{a}	65.0 ± 3.8 ^{ab}	68.8 ± 4.3 ^{a}	56.3 ± 3.3 ^{b}
Ejection rate	mL/min	−0.1 ± 0.0 ^{b}	−0.1 ± 0.0 ^{b}	−0.1 ± 0.0 ^{b}	−4.1 ± 0.6 ^{a}
Half-emptying time	min	33.6 ± 2.8 ^{a}	30.9 ± 2.5 ^{a}	26.7 ± 2.3 ^{ab}	21.9 ± 1.4 ^{b}
Refilling vol.	mL	16 ± 1.2 ^{b}	15.0 ± 0.9 ^{b}	15.3 ± 1.1 ^{b}	18.6 ± 0.9 ^{a}
Refilling rate	mL/min	0.1 ± 0.0 ^{b}	0.1 ± 0.0 ^{b}	0.1 ± 0.0 ^{b}	2.4 ± 0.4 ^{a}
Half-refilling time	mL/min	94.4 ± 2.5 ^{a}	90.5 ± 1.4 ^{a}	91.1 ± 2.0 ^{a}	90.3 ± 1.4 ^{a}
AUC	mL × 120 min	522 ± 56 ^{b}	548 ± 56.5 ^{b}	432 ± 54.3 ^{b}	1035 ± 75.8^a
Small intestinal transit					
OCTT †	min	89.5 ^{b}	80.5 ^{bc}	69.5 ^{c}	103.2 ^{a}
H2 peak †	ppm	26.5 ^{a}	21.5 ^{b}	18.5 ^{b}	22.6 ^{b}

Data are expressed as means±SEM with the exception of parameters marked with †, expressed as median values.$^{a–c}$ Within the same parameter, values with different superscript letters differ significantly ($p < 0.05$).

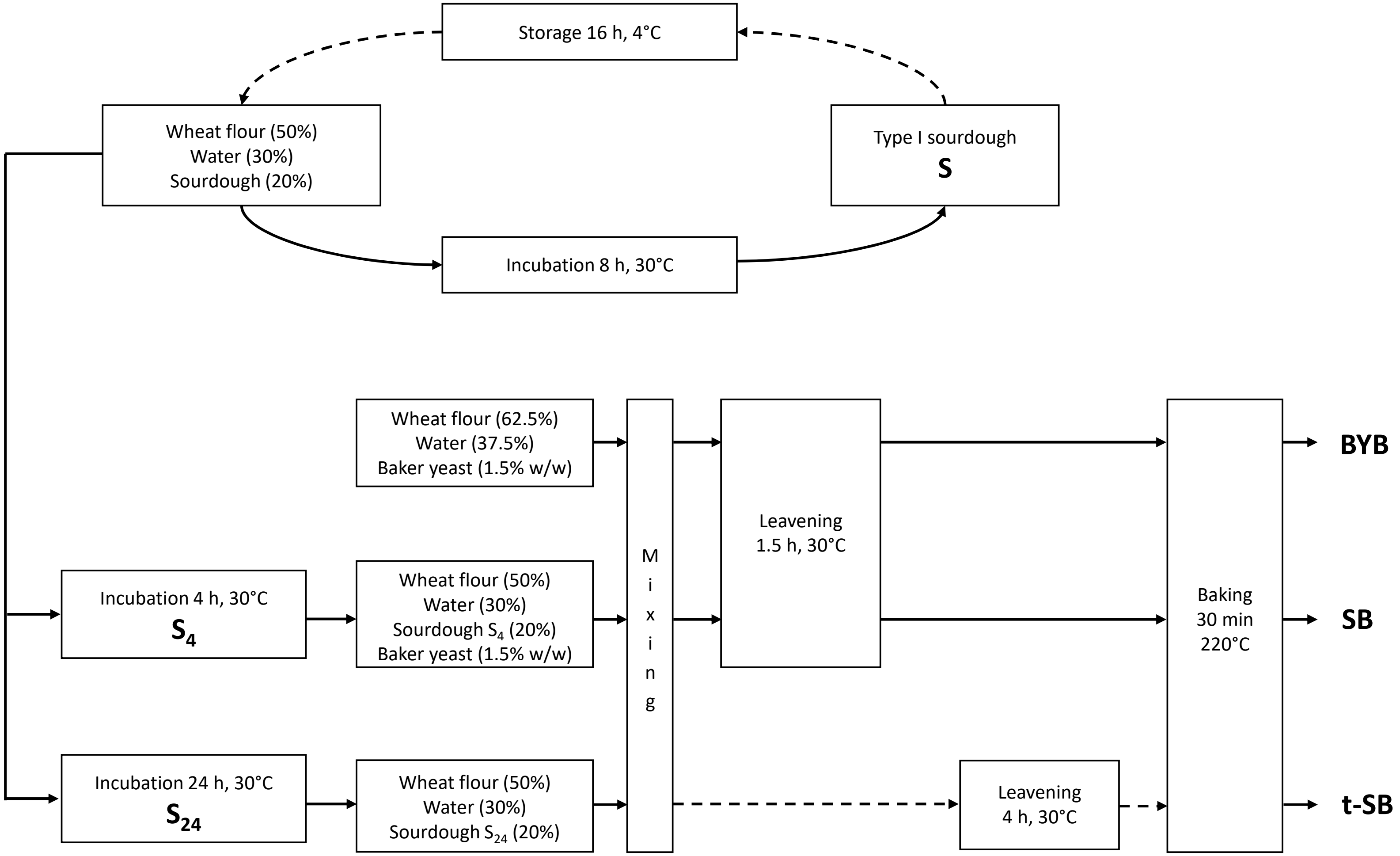

Figure S1. Flowchart of breadmaking.
